# Implementing intimate partner violence care in a rural sub-district of South Africa: a qualitative evaluation

**DOI:** 10.3402/gha.v7.24588

**Published:** 2014-09-12

**Authors:** Kate Rees, Virginia Zweigenthal, Kate Joyner

**Affiliations:** 1 Division of Public Health, Department of Public Health and Family Medicine, Faculty of Health Sciences, University of Cape Town, Cape Town, South Africa; 2 Nursing Division, Faculty of Medicine and Health Sciences, Stellenbosch University, Parow, South Africa

**Keywords:** interpersonal violence, intimate partner violence, domestic violence, mental health, health services, health systems

## Abstract

**Background:**

Despite a high burden of disease, in South Africa, intimate partner violence (IPV) is known to be poorly recognised and managed. To address this gap, an innovative intersectoral model for the delivery of comprehensive IPV care was piloted in a rural sub-district.

**Objective:**

To evaluate the initiative from the perspectives of women using the service, service providers, and managers.

**Design:**

A qualitative evaluation was conducted. Service users were interviewed, focus groups were conducted amongst health care workers (HCW), and a focus group and interviews were conducted with the intersectoral implementation team to explore their experiences of the intervention. A thematic analysis approach was used, triangulating the various sources of data.

**Results:**

During the pilot, 75 women received the intervention. Study participants described their experience as overwhelmingly positive, with some experiencing improvements in their home lives. Significant access barriers included unaffordable indirect costs, fear of loss of confidentiality, and fear of children being removed from the home. For HCW, barriers to inquiry about IPV included its normalisation in this community, poor understanding of the complexities of living with violence and frustration in managing a difficult emotional problem. Health system constraints affected continuity of care, privacy, and integration of the intervention into routine functioning, and the process of intersectoral action was hindered by the formation of alliances. Contextual factors, for example, high levels of alcohol misuse and socio-economic disempowerment, highlighted the need for a multifaceted approach to addressing IPV.

**Conclusions:**

This evaluation draws attention to the need to take a systems approach and focus on contextual factors when implementing complex interventions. The results will be used to inform decisions about instituting appropriate IPV care in the rest of the province. In addition, there is a pressing need for clear policies and guidelines framing IPV as a health issue.

Intimate partner violence (IPV) is a pervasive and complex issue that characterises partnerships worldwide. Almost 30% of women who have been in a relationship globally report having experienced physical or sexual IPV. In the World Health Organization (WHO) Africa region, this figure is 36.6% (1). In South Africa, interpersonal violence is the second highest contributor to years of life lost, after HIV (2). Of this very high burden, in women, IPV accounts for 62.4% (2), and 42.3% of working men have reported perpetrating physical violence in a relationship (3). These figures are likely to be underestimates, as the stigma surrounding IPV often leads to underreporting (4). In addition, they focus on physical and sexual abuse, and exclude emotional abuse which is less well described but appears to have a high prevalence and serious mental health implications (5).

For women experiencing IPV, negative effects span all aspects of health, from direct mortality to increasing risk factors for poor health outcomes. Mortality can be caused through homicide, or indirectly through suicide (6), maternal causes (7), and as a consequence of HIV infection (8). Morbidity could be due to multiple causes, including physical trauma, psychological trauma, and stress. In addition, the controlling behaviours of perpetrators can lead to limited reproductive control and lack of autonomy in health-seeking behaviour (1).

Recognition that IPV is an important public health concern is increasing and has recently been supported by the publication of the first WHO clinical and policy guidelines for responding to IPV and sexual violence (9). Despite this, there is limited literature describing scaled-up programmes or integrated health system responses (10).

Following the publication of a trial of universal screening for IPV that showed no improvement in quality of life or mental health outcomes (11), it appears that using a case-finding approach during health care encounters and responding in a women-centred way is likely to be of more value (12). The challenge for health systems is to integrate IPV identification and management into health services in a way that has reasonable sensitivity and addresses systemic constraints to providing this kind of care.

Many barriers to successful implementation of IPV programmes have been reported, on both provider and systems levels. Health care workers’ (HCWs) attitudes towards IPV and other reproductive services affect both women's utilisation of services and the quality of the interaction (13, 14). In another rural area of South Africa, nurses working in primary care experienced a similar prevalence of violence, and expressed similar values and attitudes about IPV, as the rest of their communities (15). Discomfort dealing with emotional issues (16) and the unrealistic assumption that women should always leave, and always want to leave violent relationships, may also affect providers’ confidence in intervening for IPV.

On a systems level, HCW concerns include lack of time during consultations (16–18), lack of training for HCWs, both prequalification and in-service (17, 19), weak referral networks (16, 17, 19), lack of confidence in management support (20), insufficient flexibility, and policy constraints (10). On a policy level, political commitment translated into clear policies and protocols is necessary for successful IPV intervention (20, 21).

In the South African primary health care system, despite the significant burden of disease, there is no standardised protocol in place for identifying or caring for IPV, resulting in generally poor recognition and inconsistent management (22). In an attempt to address this, a pilot project implementing a model for comprehensive IPV care in a rural sub-district of the Western Cape Province was undertaken between April 2012 and March 2013. The project was an intersectoral collaboration between the provincial Department of Health, Department of Social Development and the University of Stellenbosch, and aimed to integrate the intervention into the health system of the sub-district, with the intention of future expansion. This study is a qualitative evaluation of the pilot's implementation.

## Methods

### Setting

The Witzenberg is a rural sub-district of the Cape Winelands District in the Western Cape, South Africa. It had a population of 115,946 in 2011 (23). The Cape Winelands is considered to be a tourist attraction, but experiences wide socio-economic disparities. In 2010, the Witzenberg had the highest age-standardised all-cause mortality rate in the Western Cape (24). It is largely agricultural, and much of the work is seasonal, with migrant workers coming into the area during the harvest season. Rural farmworker communities in the Western Cape are generally characterised by a poor standard of living and access to services, as well as pervasive alcohol abuse. Women work in this context under particularly adverse conditions, and gendered power inequalities are further entrenched by unequal labour practices (25).

In 2012, there were nine fixed primary care facilities and one district hospital in the sub-district, as well as mobile health and community-based services. The sub-district was poorly resourced in terms of mental health services, with one mental health nurse and one full-time equivalent psychologist.

### The model

A description of the development of the piloted model has been published elsewhere (26). The first step is the identification of women experiencing IPV, using a targeted case-finding approach. The focus is on recognising cues in women presenting to primary care, for example, vague, non-specific symptoms, headaches, and mental health complaints, as well as conditions that are known to be associated with IPV, such as HIV and other sexually transmitted infections. Women are asked about violence, managed clinically, and offered referral to a dedicated IPV service.

This dedicated service was provided by a social worker employed by the Department of Social Development in the primary care facility closest to the user's home, with an intern providing back up in case of illness or annual leave. The social worker had half her time, or 10 working days a month, allocated to the pilot, which translated to one day a month being spent at each facility. The design of the pilot was to use staff already engaged in service provision, so as to assess whether the service could be implemented in other settings with similar human resource availability and burden of disease.

**Fig. 1:**
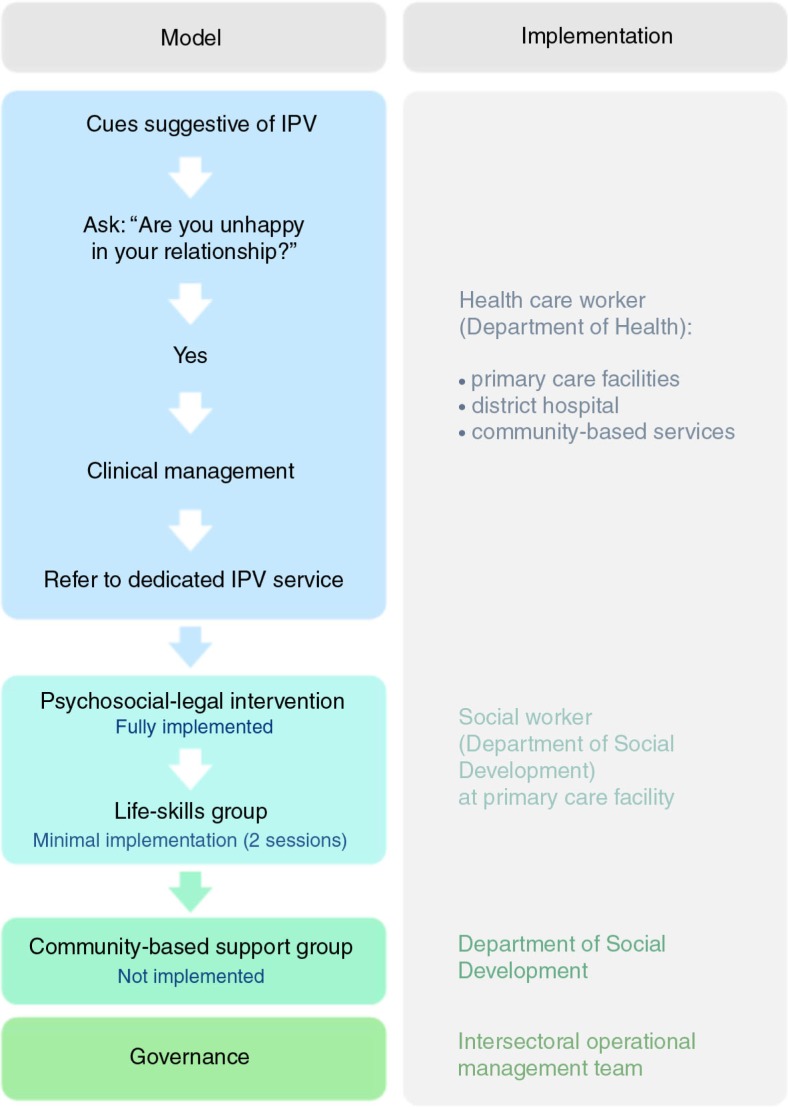
Model for IPV care implemented in the Witzenberg. Flow chart describing the model of care implemented in the Witzenberg, including service providers responsible for each step during the pilot.

The service is comprehensive, encompassing psychosocial and legal care. The first contact with the user is an assessment and intervention, and is conducted according to a protocol, covering a full history of abuse and previous attempts to access help, a safety assessment and development of a safety plan, case-finding for mental disorders (including screening for alcohol abuse), counselling, and referral to appropriate resources (see Fig. 1).

Following this contact, according to the model, users should enter a life-skills group facilitated by the same provider and covering issues relating to self-efficacy, self-care, and motivation to change. There are five sessions, whereafter community-based support groups should provide ongoing peer support, facilitated and co-ordinated by the Department of Social Development. However, during the pilot period, neither of the group phases of the model was implemented. This was largely due to a lack of buy-in for the group phases from the service providers and the implementation team, who felt that women would be afraid and uncomfortable discussing IPV in a group. There was also a lack of organisational capacity to set up groups, and a lack of experience in facilitating groups. There was no replacement protocol for follow-up, and the service provider determined whether and how users were followed up according to her usual methods of working.

An implementation team was established, consisting of district and sub-district level managers from the Department of Health, the service provider and a supervisor from the Department of Social Development, a representative of the South African Police Service, and an expert on IPV from the University of Stellenbosch. There was no overall project leader, but a responsible manager from each department. The members of the team from the Departments of Health and Social Development were jointly responsible for the implementation of the model, while the representative of the police services provided advice and support, and the University of Stellenbosch was responsible for training and technical support. Governance and strategic guidance for the pilot was provided by a district level intersectoral committee set-up to facilitate such collaborations. The implementation team held monthly meetings to address operational issues and the psychologist working in the sub-district was available to provide support to the service provider should she experience vicarious traumatisation.

Training was provided by the University of Stellenbosch. Several two-hour sessions were facilitated for HCWs, resulting in 52 nurses and nine doctors receiving training (48% of HCWs in the sub-district, achieving coverage of all facilities). The content was identification of women experiencing IPV, attitudes and misconceptions surrounding IPV, and the model and how to work with it. The social worker providing the service, as well as 19 other social workers working within the sub-district, received more extensive training over four days. This included motivational interviewing, mental health assessment, use of the protocol, life-skills and support groups.

Resources provided to the pilot consisted of the Department of Health funding for the University of Stellenbosch to provide training and technical support and the Department of Social Development allocating the service provider and her routine operational costs, for example, transport. There was no specific operational budget for the pilot, and no dedicated staffing.

### Study design

A qualitative evaluation of the pilot was conducted, aiming to understand how the model was implemented. The experience of the process by implementers, providers, intervention-users, as well as the extent of, and potential for, integration of the model into health system functions were explored.

To examine users’ perspectives, semi-structured interviews were conducted with 10 women. They were selected purposively, with HCWs identifying women who were likely to be forthcoming about their experiences, and attempting to cover a range of facilities, including those in more remote areas. Women who agreed to be interviewed were asked whether they would prefer to be interviewed in their homes or at their nearest clinic. Six women preferred to go to the clinic, where private rooms were provided, and four were interviewed during the day at their homes. When women were interviewed at home, a community health worker accompanied the interviewer, and interviews were only conducted when privacy was ensured. Community health workers are generally well accepted in the community, and the visit was framed as a follow-up to a women's health clinic visit in case of a loss of privacy.

Two focus groups were conducted with HCWs, one with primary care level nurses, and one with doctors and nurses from the district hospital, in order to explore their experiences of implementing the intervention. Only the district hospital operates 24 hours a day, and the emergency centre is responsible for seeing all trauma cases.

For the primary care focus group, the facility manager from each fixed facility involved in the intervention was invited to participate by the primary care manager of the sub-district, and to extend the invitation to other interested staff. This resulted in one representative of each fixed primary care facility (nine) attending. For the district hospital focus group, interested HCWs were invited through the nursing manager of the hospital, resulting in a group of six (two doctors and four nurses). The main reason given for not wishing to attend amongst hospital HCWs was difficulty leaving their work as the groups took place during the day. However, the intervention was focused on primary care, and was much more highly prioritised by management in those facilities, which probably led to the better response amongst primary care HCWs. They may also have seen it as an opportunity to explain their challenges with the pilot.

The differentiation of primary care and district hospital HCWs was judged to be necessary because women present to them differently and follow different pathways of care (for example, women are more likely to present to primary care with covert indications of IPV, while all trauma cases are seen at the district hospital). In addition, it was thought that the district hospital would have developed elements of its own organisational culture. However, the inclusion of both doctors and nurses in one focus group is a potential limitation, in that a power differential may exist. In general, their level of education and the status conferred to each profession by the health system is different, although their roles in this pilot were very similar. This could have led to nurses being reluctant to voice contradictory opinions. Active facilitation of the focus group encouraged the voicing of multiple views.

All members of the implementation team were interviewed. One focus group was conducted, consisting of managers from the Department of Health, with the exception of the highest level manager, with whom a semi-structured interview was conducted. This encouraged the sharing of both positive and negative viewpoints of the project team. In addition, semi-structured interviews were conducted with the social worker providing the intervention and her supervisor from the Department of Social Development, as well as the members of the implementation team from the South African Police Service and the University of Stellenbosch.

This resulted in a total of 15 interviews (10 with service users and five with implementers) and three focus groups.

Documents relating to the pilot were analysed, including initial proposals and agreements and minutes of the implementation team meetings. Monitoring data assessing the number of appointments and the number of women who received the service, as well as their characteristics, were used along with the documents, interviews, and focus groups to build a picture of the implementation process.

Data were collected in March and April 2013. The principal investigator conducted the interviews and facilitated the focus groups, in English or Afrikaans, depending on the preference of the participants. All audio recordings were translated into English and simultaneously transcribed.

Discussion schedules were used for each category of participant and data collection method. For service users, discussion schedules covered their experience of the service, including their experience of being asked about violence during a health care encounter, previous attempts to access help, expectations of the service, and benefits and harms as a result of the service. Managers and service providers were asked about their experiences of working on the pilot, including challenges and successes; how it affected daily functioning, training, and support; and experiences with intersectoral work. In addition, questions were asked about the perceived need for the intervention, and what role each department and professional should play in intervening for IPV.

### Analysis

In order to analyse the integration of the model into health systems functions, a conceptual framework developed by Atun et al. was used (27). Integration is defined as: ‘the extent, pattern, and rate of adoption and eventual assimilation of health interventions into each of the critical functions of a health system’ (27), and five key components are identified that interact to affect the adoption of interventions. These are the types of problems targeted by the intervention, the intervention itself, the adoption system (made up of multiple interconnected actors and the context within which they operate), health system characteristics, and the broader environmental context. The health system is viewed as a complex adaptive system. This pilot can be viewed as a complex intervention (less easily reproduced and needing more adaptation to integrate into local context), largely because success depends on high user and stakeholder engagement and behavioural factors.

To achieve an in-depth understanding of how the intervention was implemented, thematic analysis was used (as described by Braun and Clarke) (28). This approach was chosen for its flexibility and ability to explore patterns and underlying relationships, while preserving the influence of context (28). It involved multiple readings of the transcripts, documents, and field notes, and an exploration for themes, grouping them and looking for connections, an inductive approach. This was followed by an exploration of the data using the Atun et al. conceptual framework described above (27), a deductive approach. All data were iteratively coded and a code diary was kept. Data were organised using Open Code software (29). Triangulation of data from all sources and respondents allowed a unified understanding to be developed, incorporating the viewpoints of all role-players. Contradictory data were purposely sought and examined to improve the trustworthiness of conclusions. Reflexivity was encouraged using field notes and a research diary throughout the evaluation. Final themes were based on the conceptual framework and modified in order to adequately describe the user perspective and integrate inductively generated themes.

### Ethical considerations

Ethical approval was obtained from the University of Cape Town, Faculty of Health Sciences Human Research Ethics Committee (reference 655/2012) and permission to conduct the research was granted by the Department of Health. Written informed consent was obtained from all participants, in English or Afrikaans, according to their preference.

## Results

In the 11 months during which the intervention was provided, 165 women had appointments for the service, and 75 women received the initial intervention according to the protocol. Only 45% of those who were referred and successfully made an appointment attended. No community-based support groups took place, and only the first of the five life-skills group sessions was facilitated at two venues. These numbers were felt by the implementation team to be low, and raised questions about whether this was a justifiable use of the service provider's time. Over the same period, the police services of the largest town in the sub-district recorded 373 domestic violence complaints.

The characteristics of users are presented in Table 1. The median age of users was 32 years, ranging from 16 to 58 years, and severe abuse was experienced, with women reporting on average nine different forms of abuse. Eighty-two percent of women were scored as being at high or severe risk of injury or death. Forty-eight percent had not previously accessed legal assistance, and 65% were referred for further mental health assessment.

**Table 1:** Characteristics of service users

Characteristics of service users during pilot		
Age (median, IQR)	32 (25, 41)	
		Percentage (frequency)
Relationship to abuser	Married	34 (22)
	Cohabiting	41 (26)
	In a relationship	11 (7)
	Previous relationship	14 (9)
Types of abuse	Physical abuse	89 (58)
	Emotional abuse	88 (57)
	Sexual abuse	62 (40)
	Financial abuse	51 (33)
Frequency of abuse (2 years)	More than 20 times	40 (20)
	10 to 20 times	24 (12)
	Less than 10 times	36 (18)
Safety score	High risk	54 (35)
	Severe risk	28 (18)
Previous legal help	Protection order	34 (22)
	Charge laid	48 (31)
	Neither	48 (31)
Referrals to mental health	Total	65 (42)
	Suspected depression	83 (35)
	Suspected anxiety	19 (6)
	Suspected PTSD	14 (8)
	Suspected alcohol abuse	19 (8)
	Suspected substance abuse	5 (2)
	Multiple suspected diagnoses	33 (13)

Ten women were interviewed in an attempt to understand how users experienced the intervention. They were spread over six different primary care facilities. Compared to the rest of the service users, they had similar demographic characteristics and the abuse they experienced was of a similar severity. However, more of the interview participants had previously accessed legal help and were referred for further mental health assessment. These participants are described in Table 2.

**Table 2:** Profile of interview participants

Interview participants (*n*=10)		

	Median	IQR
Age	35	30, 43
Months since intervention	6	5, 7
		Frequency
Interview site	Clinic	6
	Home	4
Clinic at which intervention was received		
	Nduli	2
	Tulbagh	2
	Breerivier	2
	Op Die Berg	2
	Prince Alfred Hamlet	1
	Bella Vista	1

Analysis of these interviews, as well as the focus groups and interviews with managers and service providers, resulted in six final themes: environment, user experience, access barriers, health system influences, intervention characteristics, and attitudes to IPV.

### Environment

Both users and HCWs described living in an environment permeated by violence. Users experienced physical, emotional, financial, and sexual abuse at the hands of their partners, as well as their partners’ families or their own family members. They described living in fear and anger, in some cases leading to the perpetration of acts of violence against their partners. High levels of alcohol misuse were perceived to be associated with IPV and family and community violence.

Because as soon as I am sober, then [he] upsets me. He comes to me with his drunk things, and says things and how do I take it? … He doesn't hit me when he's sober. He doesn't mess with me. But if I'm drunk and he's drunk … (Interview 15)

IPV was understood to be a social norm, with traditional gender roles holding sway. Women had very little autonomy, particularly over sex in their relationships. They were deeply disempowered socially and economically, and were expected to fulfil the roles imposed by a patriarchal society.

Because he still hurts me every day. And when he's at home I must play the darling wife, I must do what he wants and I must sleep with him … I am really trying my best to be a wife to him and respect him in everything he does … And then people come from outside and they say, wash his clothes, show him how you feel. You are still his wife, wash and iron his clothes. Get his food ready. (Interview 5)

### User experience

Participants appreciated having someone listen to them and share the weight of their problems.

But I know she can listen to all my problems that I have. I don't have to bottle it up. I don't have to sit every time and think, oh, what must I do now so he can stop shouting at me like that? (Interview 3)

When I was done talking to her, everything was alright again. She understood me and I understood her … It went very well. We spoke like people who knew each other … when I was in conversation with her, everything disappeared from me. (Interview 8)

The understanding, support, and validation they felt they received was of increased importance in the context of isolation imposed by the controlling behaviour of abusers, the feelings of shame associated with abuse, and the constant negative input of emotional abuse.

What was in my heart, I poured it all out … and I felt like a person again. (Interview 4)

I felt that which she gave me healed me a bit afterwards. (Interview 9)

The intervention and the positive experience of being heard and supported led to improvements in communication within relationships, or with children, although temporary in some cases.

In my house I changed a lot of things, because I feel me and my children are much closer to each other than we were. And we can talk to each other especially me and my eldest daughter, we can talk openly … (Interview 13)

No harms were reported as a consequence of the intervention and there was variation in whether partners were informed of the reason for the visit. However, confidentiality was an overriding concern, particularly relating to the clinic environment.

That time a lot of one's personal things leaked here. That is why I am very cautious when I come to the clinic. I will not easily walk in here and go and talk to a sister because I know what has happened before. (Interview 14)

### Access barriers

Access can be conceptualised as the degree of fit between health systems and users across three dimensions: availability, affordability, and acceptability (30). Access barriers were identified in each of these domains, either relating to access to health services in general, or to this intervention in particular.

Availability of the intervention was very limited in that it was provided only once a month at each fixed primary care facility. This was due to a lack of resources, with one service provider having 10 days a month dedicated to the intervention, spread over a large geographical area. The timing of the intervention was problematic for the same reason, with women having to wait up to a month from the time of their referral. This is likely to have affected their motivation and readiness to attend.

Although primary health care is free in South Africa, indirect costs, including transport and loss of income, made the intervention unaffordable to many women. Seasonal employment is common in the area, and workers are not paid for time off to attend clinic appointments.

Because you don't get paid if you don't come to work. You can be sick and come to the clinic, but you don't get paid … I feel at least I'm working, and it helps me to earn a few cents for the two boys and the girl. (Interview 4)

Threats to acceptability included fears that children would be removed from their mother's care as a result of any interaction with a social worker. Women feared social workers would take the children away from the home if violence or drinking was disclosed. This led to reluctance to attend appointments.

A lot of times people told me your child will be taken away and all those things … The only thing I had in the back of my mind, I just waited for the moment they will take away my child. And then I told her everything, and then she explained to me what she came to do. She also said she isn't coming to take away the children. (Interview 8)

Confidentiality was an important concern in light of the small communities to which people belong and the stigma associated with IPV. Participants feared their partners would find out about the visit either through a breach of confidentiality or a community member seeing them at the clinic's IPV service. An associated fear was that visiting a social worker would identify them as having social or mental health problems.

Finally, there was a misconception amongst both users and HCWs that the intervention was largely about legal redress for IPV. This has previously been the dominant response of the health services, and women were wary that they may be pressurised to lay a charge against their partners if they attended.

### Intervention characteristics

The support and resources required to implement an intersectoral intervention having this level of complexity were underestimated at a strategic level. The motivation to work intersectorally stemmed not only from the recognition of a joint mandate but also from a desire to share resources and capacity in an under-resourced environment. The intersectoral nature of the implementation team led to additional complexities both in terms of the structure of the intervention and the relational issues between partners at various levels. The scale of the intervention (service provision through multiple service delivery platforms in a large geographical area) further added to these challenges.

It's the first real collaboration of this nature between health and the various other departments. So it takes a lot of other softer issues that we don't normally deal with, and having to iron that out takes a lot of effort and energy. (Focus group 1)

In the planning phases, the intersectoral team failed to adequately clarify roles. During the process of adapting the model for implementation, the local implementation team felt that they had not been adequately consulted and had been allowed insufficient flexibility. An important example of this was the group phases of the original model, which the local team considered to be inappropriate in this setting. However, the model was not adjusted, and despite the fact that very few groups took place (largely due to this resistance), an alternative mode of delivery was not instituted during the pilot period. A change in local managers responsible for the pilot at this crucial stage contributed to divisions during the adaptation process, and may have negatively impacted ownership. Further, engagement between the implementation team and the service providers (both HCWs and social workers) was absent. This led to a lack of trust and resistance from service providers in the initial phases. When attempting to provide supervision to service providers, complications regarding rigid management hierarchies and communication challenges led to the formation of alliances, further decreasing trust. At a service delivery level, this may have negatively impacted the quality of the intervention, as service providers were not as receptive to the ongoing training and mentoring that was offered as they could otherwise have been.

At a strategic level, not all partners were adequately represented on the implementation team in terms of decision making power. In addition, the mandate from higher management structures to implement the pilot, as well as ongoing levels of support for the intervention varied.

A dominant theme was a desire for services to work with men. Participants recognised that those perpetrating the abuse have a lot of ‘stress’ and difficulty communicating, in particular their own emotions. Alcohol and substance abuse were also identified as major underlying factors needing to be addressed. In addition, offering help to women experiencing abuse and not the perpetrators was interpreted by women as neglecting to address the cause of the problem. This mirrored the sentiments of the HCWs and the managers, who expressed concern that the intervention focused on the victim rather than the perpetrator.

It's only the wife we are talking to, we never talk to the husband as well; so you don't explain to the husband ‘we realise there is a problem in your house how can we fix it?’ And I think that is a very big gap at the end of the day. (Focus group 3)

### Health system influences

Participants in this study identified that IPV is likely to require more than one user–provider interaction, and that continuity of care would be crucial in providing appropriate care over a sustained time period. The South African primary health care system has historically been geared towards acute episodic care (31), and continuity of care remains a challenge. The primary health care system is also geared to curative care, and HCWs usually do not have the counselling skills needed to facilitate behaviour change.

Other system level barriers to implementation existed. Inefficient referral systems often put the onus on the user to make appointments which may have required taking time off work or making expensive cellular phone calls. There was also a lack of time in the consultation to introduce subjects that may lead to difficult and lengthy discussions, and multiple things to remember in the context of comprehensive care led to HCWs forgetting to inquire about IPV. Mental illness and social problems are also stigmatised, and privacy is difficult to maintain due to infrastructure and systems constraints. Confidentiality is a concern for users, and participants expressed fears that confidentiality would be lost, either through HCWs or community members who may have witnessed them attending this service.

The role of the health system in addressing IPV was dominantly understood to consist of identifying women experiencing IPV and linking them to further services, but not taking primary responsibility for their care. This was consistent with how this pilot was implemented, as social workers were responsible for comprehensive (excluding medical) care. However, the ingrained perception that IPV is not a health problem is likely to have impacted negatively on the integration of inquiry about violence into routine HCW functioning.

Because the actual bigger body of the whole thing lies with the counselling, and that's the social worker's role. The bigger role is definitely with the social worker and not with health. Health, definitely to identify and to refer … but the core function lies with the social worker. (Focus group 1)

The reciprocal perception from the user perspective that the health services would not be an appropriate place to discuss emotional or social problems, or that HCWs would respond only by directing users to legal interventions, appears to have been a significant access barrier.

Lack of experience on the part of the social workers as well as a perceived lack of commitment, due at least in part to resource constraints (for example, difficulties accessing transport and telecommunications), led to decreased levels of confidence in the intervention from HCWs. Booking women for the service who subsequently did not come, a sense of futility about intervening in IPV, and frustration generated when women not leaving violent relationships was interpreted as a failure of the intervention, compounded this lack of confidence and impacted negatively on referrals.

Because they have to go back to the same circumstances. They need their partners … We try to book the cases that come repeatedly but then they just don't show up … (Focus group 3)

The professional values of service providers have previously been found to be important in providing IPV care (26). Similarly, in this pilot, it was found that complete implementation would have required exceptional commitment, particularly as the social workers had to advocate for a new service while working in the health system for the first time. Other factors that affected the capacity of the social workers included a high concurrent case load, lack of management support, and organisational limitations. Limited mental health knowledge and skills, and viewing mental health as outside of their scope of practice, led to reluctance to tackle the mental health aspects of the intervention, and additional training and mentoring were required to ensure this was done adequately.

Support from a psychologist, attempting to mediate the effects of vicarious traumatisation for service providers, was offered but not taken up, suggesting that this type of support needs to be provided in a more structured manner.

### Attitudes to IPV

High levels of violence experienced in this community and widely accepted traditional gender norms have led to some HCWs accepting that IPV is a normal part of life. There was an underlying lack of understanding of the complexities of living with violence and trying to leave a violent relationship. The gendered aspects of IPV were often overlooked. IPV as a health condition was defined according to the severity of abuse, and whether HCWs felt that users’ situations warranted referral. Users missing appointments was also interpreted as an indication that they did not need or desire the service.

Then I would ask them, but why did you not refer? And they would say to me, but you know, that has been happening for so long … And some of them will even say to me, but you know, they wanted it or they asked for it. Something happened and she actually made her husband angry, so it isn't really intimate partner violence … so it's actually okay, so why refer? (Focus group 1)

In the district hospital, HCWs were so used to violence that women presenting with assault by a partner were regarded as a normal occurrence and not singled out for further psychosocial management.

## Discussion

This pilot represented an attempt to integrate a complex intervention for comprehensive IPV care into a rural district health system, which is not well suited to the care of chronic conditions, lacks mental health resources, and has numerous barriers to access. IPV is a phenomenon with complex social and structural roots. Poverty, gender inequality, and alcohol misuse are entrenched in the Witzenberg, and women are more vulnerable to exploitation in this agricultural community than their (already socioeconomically disempowered) male counterparts. For an intervention to have a significant impact, the stigma surrounding IPV as well as underlying values and attitudes to gender would have to be transformed, amongst both service providers and community members.

User experiences of the intervention were overwhelmingly positive, in some cases leading to improvements in their home lives. WHO guidelines recommend that a women-centred approach be adopted when responding to IPV (9). Literature on women's expectations and experiences of health services shows that they want health care providers to be non-judgemental, empathic, and understanding, and to provide validation (32–34), and that when these features are absent, the encounter can be damaging rather than helpful (35). In a South African study, the service women reported wanting most often was counselling (4). Users described experiencing the approach of the intervention as consistent with these guidelines. They felt understood, supported, and validated and appreciated being listened to. In the context of poor social support and imposed isolation, these features were valued.

The guidelines further recommend assisting women to access information and resources, assisting them to increase safety, and providing or mobilising social support (9). Referrals to mental health services in this pilot were high (although referral pathways were not always effective) but facilitating access to other resources was less successful. Referral networks both within the health system and between other agencies need to be strengthened to support continuity and allow women access to further community resources. A lack of structured follow-up also contributed to gaps in the continuity of care. The high number of mental health referrals is consistent with a previous South African study that found 66.4% of women obtaining protection orders against their partners to have severe depression symptoms, and 51.9% to have severe PTSD symptoms (36). It also highlights the importance of mental health skills and experience in IPV care providers.

Both users and implementers expressed a desire for services to work with men, both because they were perceived to need psychosocial support, and in addressing violence in the home. How to intervene with men should be considered, in the context of health services that are often not appropriately geared to meet men's needs, as well as prevailing constructions of masculinity negatively influencing their utilisation of health services (37).

A significant number of domestic abuse cases were reported to the police services of the largest town in the Witzenberg during the time period of the pilot. These cases represent women actively seeking help for IPV, albeit not from the health services, and led to the implementation team viewing the number of users generated by the pilot as inadequate. Expectations that, because levels of violence are high in the area, women would be readily identified proved unrealistic, because of the complex social and structural factors underlying IPV, as well as the health system constraints.

Reasons for low referrals to the intervention and low attendance amongst those who were referred included access constraints that affect health services more generally and specifically relating to the service, as well as provider, system, and societal level barriers to HCWs inquiring about IPV.

Availability of the intervention was limited, and the costs of missing work or finding transport often made it unaffordable. Key threats to acceptability included a lack of trust in the confidentiality of the health services, often cited in the literature as a barrier to disclosing IPV (32, 34, 38), as well as a fear that disclosure would lead to social workers removing their children from the home.

Important barriers to HCWs inquiring about violence included the normalisation of IPV leading to HCWs giving the intervention a low priority. Access to reproductive services is significantly affected by HCWs attitudes (14), and whether and how they inquire about IPV is crucial to successful intervention, despite the dedicated IPV service being provided in a manner acceptable to users.

Poor recognition that IPV is a valid health problem, and the perception that the health system plays a limited role in providing IPV care, also affected attitudes towards the intervention. Coherent national and provincial policy frameworks are needed to begin to shift these views, furthering the efforts of the WHO in publishing clinical and policy guidelines which clearly frame IPV as a health issue.

The piloted model allows for integration of services from the perspective of the Department of Health, with the primary care provider inquiring about IPV and providing initial medical care and referral. This was not fully achieved, however, and the dedicated IPV service was not integrated into routine service delivery, or other health system functions such as training and governance. There is no consensus that interventions targeting specific health problems should always be fully integrated (39), but the current reengineering of the primary health care system towards comprehensive primary care suggests that integration would lead to better sustainability. In addition, participants in management roles expressed that integration of services is a priority for them, and WHO guidelines recommend that IPV services be as integrated as possible (9).

The challenging nature of working intersectorally was highlighted during this pilot, particularly relating to differing levels of management support, decentralisation of control and availability of resources, as well as a lack of clarity regarding partners’ functions. The formal structures of intersectoral action were found to be important. However the effects of informal relationships and communication, as well as shared ownership and understanding, were more significant. Ultimately the formation of alliances proved destructive.

A contradiction became apparent between the recognised need to deliver integrated services through intersectoral platforms, and the tight parameters within which managers and service providers are required to operate. The theory of professional closure, describing the carving out of exclusive professional definitions to create increased status or reward, can be applied to interactions between the various professionals involved in this intervention and their power dynamics (40). Considering the development of professions in this light adds to an understanding of the difficulties inherent in working intersectorally.

### Implications

The barriers to implementation described above require that a health systems approach be taken in considering scale up of this model, interrogating how all elements of the health system would be affected by implementation. In so doing, it could attempt to strengthen referral systems, continuity of care, HCW skills, and platforms for intersectoral action. A high degree of flexibility is required, allowing adaptation to local context and resources, and engagement processes to ensure buy-in from all partners are crucial in the planning phases. The service should be integrated into health system functions as far as possible in order avoid an unsustainable vertical service. In addition, the pervasiveness of alcohol and its links to violence highlight the need for a multifaceted approach to providing care for IPV.

Longer term evaluation of this intervention is needed to examine user outcomes and determine its effectiveness, and also to assess the effects of additional time on implementation. There is a need for appropriate services for women presenting to the primary health care system who are experiencing IPV, as well as policies and protocols guiding these services, but the resource and management requirements for implementation should not be underestimated.

### Limitations

This evaluation did not examine outcomes, so the effects of the intervention on violence, quality of life, and mental health measures are unknown. Processes and context were explored, which will necessarily vary in different settings, limiting generalisability. However, health system barriers to providing IPV care are likely to be similar in similar settings. In addition, women who either declined the intervention or did not attend their appointments were not interviewed, so their perspectives were missed. It is very possible that other access barriers would have been identified had this not been the case.

Interviews were conducted in English or Afrikaans, and all analyses undertaken in English. Translation was therefore necessary, with the potential loss of nuanced meaning from the data. In an attempt to avoid this as much as possible, simultaneous transcription and translation was used.

The principal investigator in this study was employed by the Department of Health. This may have impaired participants’ ability to answer certain questions critically. In terms of the users, social desirability bias may have been introduced. However, it may also be viewed as a strength as it allowed a fuller understanding of the organisational context within which the pilot was implemented.

## Conclusion

This study evaluated the process of implementing a model for comprehensive IPV care in a rural sub-district of the South African district health system. It was an ambitious undertaking, requiring system-wide implementation, multiple stakeholders, and external training, while fundamentally challenging entrenched value systems of privilege and power. Contextual factors such as high levels of alcohol abuse and the double exploitation of women in this farming community added to these challenges and point to the need for multifaceted approaches to addressing IPV.

The pilot model was not fully implemented in that the group phases did not occur, and was hindered by barriers to inquiry about IPV (evidenced by low referral numbers) as well as by access barriers, including those limiting acceptability (evidenced by a low proportion of women keeping appointments). The value of a qualitative process evaluation has been demonstrated, and the findings will be used to inform decisions about instituting appropriate IPV care in the rest of the province.
